# Supplementary material for: Direct visualization of edge state in even-layer MnBi2Te4 at zero magnetic field
Source: Nat Commun. 2022 Dec 13;13:7714. doi: 10.1038/s41467-022-35482-0 (PMC9747779; doi:10.1038/s41467-022-35482-0)
Supplement: Supplementary file 1 — Supplementary Information [file 41467_2022_35482_MOESM1_ESM.pdf]

Supplementary information

Direct visualization of edge state in even-layer $MnBi_2Te_4$ at zero magnetic field

Weiyan Lin[1†], Yang Feng[2†], Yongchao Wang[3,4†], Jinjiang Zhu[2], Zichen Lian[3], Huanyu Zhang[2], Hao Li[5,6], Yang Wu[6,7], Chang Liu[3,8], Yihua Wang[2,9], Jinsong Zhang[3,10], Yayu Wang[3,10], Chui-Zhen Chen[11,12], Xiaodong Zhou[1,13,14*] and Jian Shen[1,2,13,14,15*]

[1]State Key Laboratory of Surface Physics and Institute for Nanoelectronic Devices and Quantum Computing, Fudan University, Shanghai, China.

[2]Department of Physics, Fudan University, Shanghai, China.

[3]State Key Laboratory of Low Dimensional Quantum Physics, Department of Physics, Tsinghua University, Beijing, China.

[4]Beijing Innovation Center for Future Chips, Tsinghua University, Beijing, China.

[5]School of Materials Science and Engineering, Tsinghua University, Beijing, China

[6]Tsinghua-Foxconn Nanotechnology Research Center, Department of Physics, Tsinghua University, Beijing, China.

[7]Department of Mechanical Engineering, Tsinghua University, Beijing, China.

[8]Beijing Academy of Quantum Information Science, Beijing, China.

[9]Shanghai Research Center for Quantum Sciences, Shanghai, China.

[10]Frontier Science Center for Quantum Information, Beijing, China.

[11]School of Physical Science and Technology, Soochow University, Suzhou, China.

[12]Institute for Advanced Study, Soochow University, Suzhou, China.

[13]Zhangjiang Fudan International Innovation Center, Fudan University, Shanghai, China

[14]Shanghai Qi Zhi Institute, Shanghai, China.

[15]Collaborative Innovation Center of Advanced Microstructures, Nanjing, China.

[†] These authors contributed equally to this work

[*] Emails: zhouxd@fudan.edu.cn, shenj5494@fudan.edu,.cn

Supplementary Note 1: thickness determination of few-layer $MnBi_2Te_4$ devices

The exact thickness of $MnBi_2Te_4$ thin flakes is determined by checking its optical reflectance and following an empirical method we have obtained. Supplementary Fig. 1(a) displays an optical image of few-layer $MnBi_2Te_4$ flakes cleaved onto SiO_2/Si substrate. Image was taken in reflection mode before PMMA coating and other fabrication process. We first determine the thickness of areas with different colors by Atomic Force Microscope (AFM, Bruker Innova). Number of SLs is labeled on selected flakes in Supplementary Fig. 1(a). We then calculate the optical image contrast between substrate and selected flakes by extracting the RGB value from the selected flakes and substrate in the original optical image, and dividing the difference between them by the substrate background. We plot such a contrast between substrate and flakes as a function of the number of SLs in Supplementary Fig. 1(b). We have performed this AFM/optical measurement on multiple $MnBi_2Te_4$ devices, and the resultant data are all plotted in Supplementary Fig. 1(b). We find a good one-to-one correspondence between the optical image contrast and the number of SLs. This relation serves as an empirical method for us to determine the thickness of $MnBi_2Te_4$ flake.

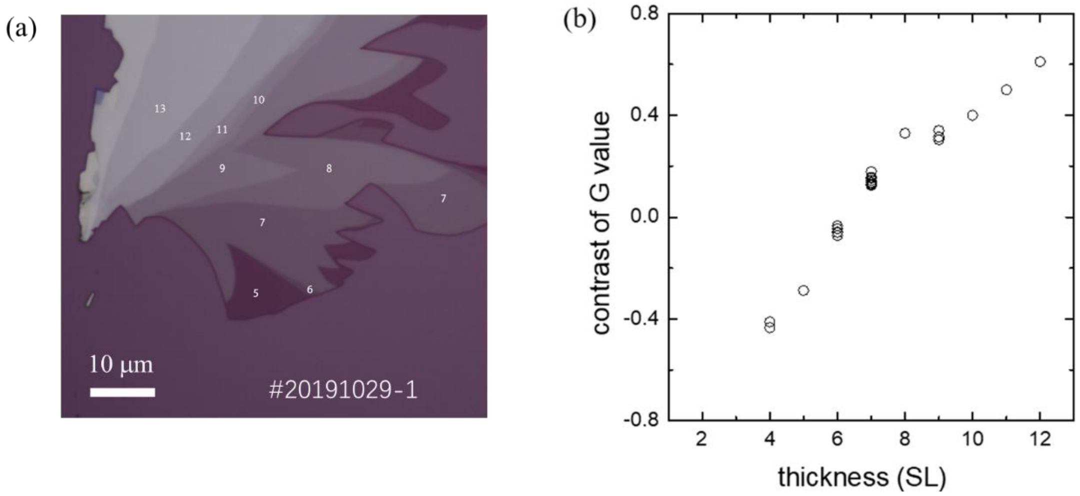

Supplementary Fig. 1 (a) Optical image of few-layer $MnBi_2Te_4$ flakes cleaved onto SiO_2/Si substrate. Image was taken in reflection mode before PMMA coating and other fabrication process. Number of SLs is labeled on selected flakes as determined by AFM. The scale bar is 10 μm. (b) The optical image contrast as a function of the number of SLs.

Supplementary Note 2: sSQUID characterization of few-layer $MnBi_2Te_4$ devices

The A-type AFM order of bulk $MnBi_2Te_4$ makes few-layer $MnBi_2Te_4$ flakes with even- or odd-layer thickness to display drastically different topological properties due to their different net magnetization values. Recent controversies regarding the even-odd layer thickness determination in $MnBi_2Te_4$ flakes call for a better approach to identify this property[1]. Here we adopt a scanning superconducting quantum interference device (sSQUID) to directly measure the static magnetic flux generated by net magnetization of the sample. Being an ultra-sensitive probe of magnetization, sSQUID provides a powerful solution to differentiate even- from odd-layer $MnBi_2Te_4$ flakes.

Supplementary Fig. 2(a) shows the optical image of our $MnBi_2Te_4$ device in a large sample area which includes the 6-SL Hall bar region and a thicker region. Supplementary Fig. 2(c) displays the sSQUID flux image of the same area as Supplementary Fig. 2(a). Two flux signal levels are easily identified corresponding to odd- and even-layer $MnBi_2Te_4$ due to its special A-type AFM order. Shown in Supplementary Fig. 2(b) is the flux line-cut profile along the green line in Supplementary Fig. 2(c) across the 6-SL region and the thicker region. The flux signal level of 6-SL region is roughly 25% of that of the thicker region, justifying the even-layer (odd-layer) assignment to the 6-SL region (thicker region). The residual flux signal in 6-SL region is due to an imperfect cancellation of magnetization which may come from the surface reconstruction of magnetization. Note that Supplementary Fig. 2(c) was obtained at zero field after an initial magnetization at +9 T. One can magnetize the sample at -9 T which should lead to opposite flux signal. This is what we observed in Supplementary Fig. 2(d) showing the sSQUID flux image after the initial magnetization at -9 T. In short, our sSQUID characterization unambiguously confirms the even-layer property of our 6-SL $MnBi_2Te_4$ device.

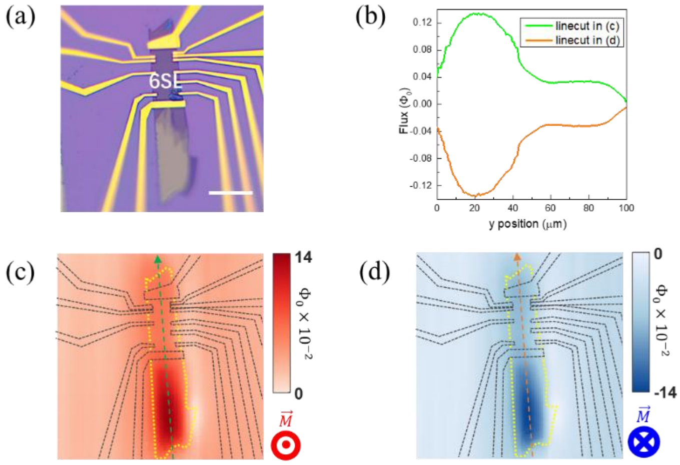

Supplementary Fig. 2 (a) Optical image of the $MnBi_2Te_4$ device. The scale bar is 20 μm. (b) Flux line-cut profiles across the 6-SL region and the thicker region extracted from (c) and (d). (c,d) The corresponding sSQUID flux images after a (c) positive and (d) negative initial magnetization.

Supplementary Note 3: gate voltage and field dependent transport characterization of 6-SL $MnBi_2Te_4$

We provide more gate voltage and field dependent longitudinal resistance R_{xx} and Hall resistance R_{yx} of the 6-SL $MnBi_2Te_4$ device presented in the main text. Supplementary Fig. 3(a) is the optical image of the Hall bar device with electrodes properly labeled in the schematic of Supplementary Fig. 3(b). R_{xx} and R_{yx} of Fig. 1(c) in the main text is measured between electrodes B and C, and between electrodes H and C, respectively. Supplementary Fig. 3(c-f) show the same R_{xx} and R_{yx} under more gate voltage and magnetic field conditions. This transport characterization clearly shows that a Chern insulator phase is only realized when the magnetic field is larger than 6 T and when the bulk is gated to charge neutral point (CNP) with gating centered at around +33 V. At CNP, a zero Hall plateau (ZHP) is observed at small fields with a large longitudinal resistance. Away from CNP, one sees a change from p-type to n-type transport from the overall slope of the Hall trace due to the Fermi level shift with gating.

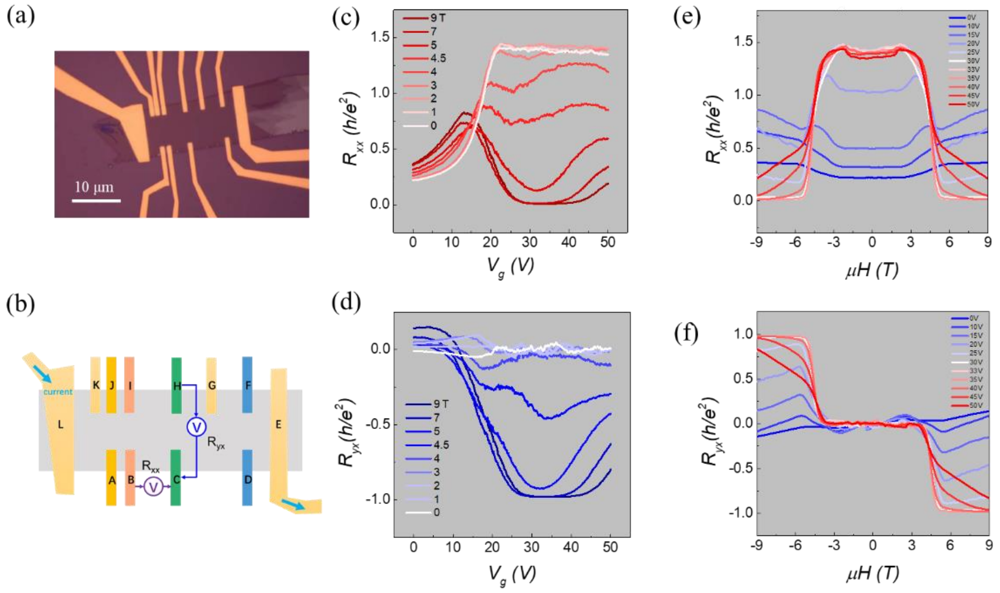

Supplementary Fig. 3 (a) The optical image of the device. The scale bar is 10 μm. (b) Transport measurement configuration. (c) Gate voltage dependent R_{xx} at several discrete fields. (d) Gate voltage dependent R_{yx} at several discrete fields. (e) Field dependent R_{xx} at several discrete gate voltages. (f) Field dependent R_{yx} at several discrete gate voltages.

Supplementary Note 4: the uniformity of ZHP phase and its in-gap edge state in 6-SL $MnBi_2Te_4$

We demonstrate the uniformity of ZHP phase and its associated in-gap edge state in our device to rule out disorders-induced spatial variations in our sample. Supplementary Fig. 4(a) shows the optical image of the 6-SL $MnBi_2Te_4$ device with all the electrodes clearly labeled. Supplementary Fig. 4(b) presents the Hall resistance R_{yx} of 4 pairs of opposite electrodes taken at +33 V gate voltage. Note that these are raw data before antisymmetrization. They all show a ZHP with negligible variations among them. In Supplementary Fig. 4(c), we display the zero field sMIM images at 0 and +40 V gate voltages which was taken at the red rectangular area denoted in Supplementary Fig. 4(a). One sees a uniform metallic or insulating bulk when the Fermi level is set to the valence band or the band gap. A uniform conductive edge state is also observed. These data demonstrate the high sample quality of our 6-SL $MnBi_2Te_4$ device.

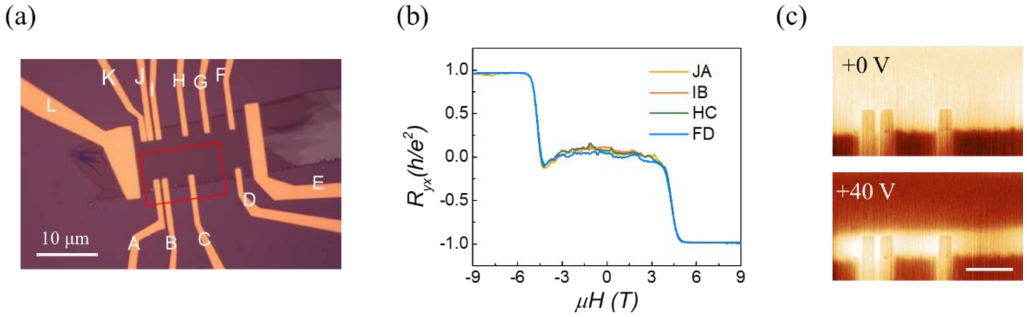

Supplementary Fig. 4 (a) The optical image of the device. The scale bar is 10 μm. (b) Hall resistance R_{yx} as a function of magnetic field for 4 pairs of opposite electrodes. (c) sMIM images of the red rectangular area denoted in (a) taken at zero field and 0 and +40 V gate voltages. The

scale bar is 3 μm.

Supplementary Note 5: bulk transport measurement of 6-SL $MnBi_2Te_4$

To probe the metal-to-insulator transition (MIT) of the bulk in global transport measurement, a special experimental set-up is adopted as shown in Supplementary Fig. 5(a). We basically run a current through a pair of electrodes on opposite site of the device while grounding all other electrodes. While most of the injected current will flow through the edge to the ground due to the existence of conductive edge state, current that goes through the bulk can be measured at the drain electrode as I_{bulk} which reflects the bulk resistance state. The field dependent I_{bulk} is presented in Supplementary Fig. 5(b) indicating the bulk MIT transition with the change of bulk current by an order of magnitude. We can also measure the voltage drop between source and drain, i.e., V_{HC} in our case, then the bulk resistance can be estimated as $R_{bulk} = V_{HC}/I_{bulk}$ as shown in Supplementary Fig. 5(c). The bulk resistance at the metallic state is lower than 1 MΩ. One can thus estimate the edge state conductivity to be at the same level (~1 $\mu s/\square$) because the metallic bulk shows similar sMIM signal level with the edge (Fig. 3a).

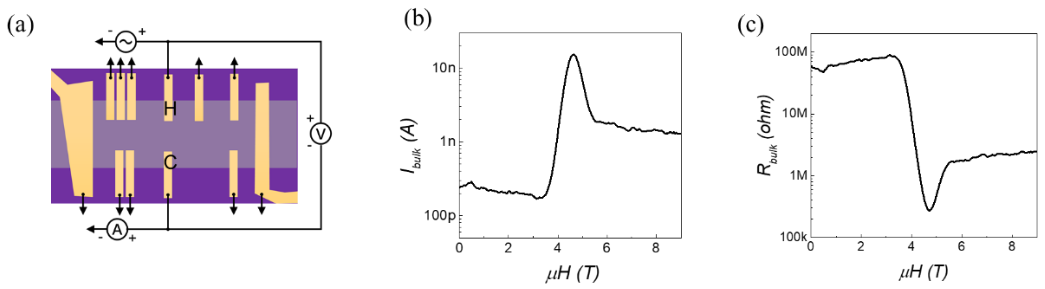

Supplementary Fig. 5 (a) The transport set-up for bulk resistance state measurement. (b) The field dependent bulk current I_{bulk} taken at +33 V gate voltage. (c) The field dependent bulk resistance R_{bulk} taken at +33 V gate voltage.

Supplementary Note 6: nonlocal transport measurement of 6-SL $MnBi_2Te_4$

A nonlocal transport measurement is performed on 6-SL $MnBi_2Te_4$ to reveal the nature of its charge transport. Supplementary Fig. 6(a) shows the experimental configurations of current and voltage electrodes. The resistance value Rmn,pq = Vpq/Imn is obtained from the voltage difference between electrodes p and q divided by the current flowing from electrodes m to n. Supplementary Fig. 6(b) displays the nonlocal resistance $R_{12,34}$ as a function of magnetic field taken at +33 V gate voltage when the Fermi level is tuned within the band gap. The large nonlocal signal at low field regime corresponding to ZHP phase strongly suggests the existence of edge conduction with dissipations at zero field. Such signal cannot be attributed to the bulk states which exponentially decays with the distance from the current electrodes. Nor can it be attributed to disorders which is not expected to carry currents. Moreover, the vanishing nonlocal signal at high field regime corresponding to Chern insulator is due to the dissipationless chiral edge state.

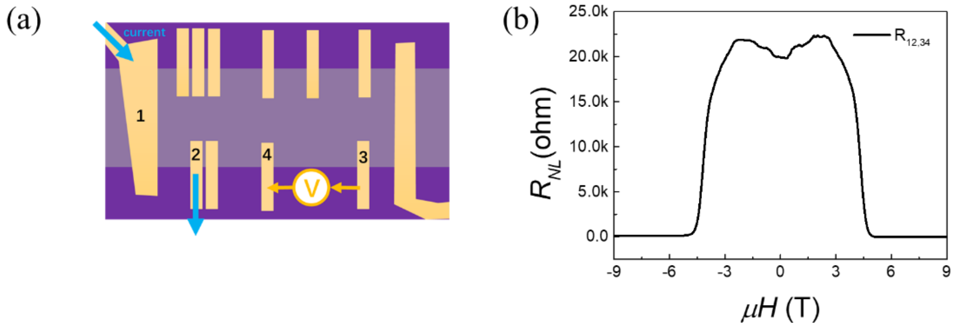

Supplementary Fig. 6 (a) The nonlocal transport measurement set-up. (b) Field dependent nonlocal resistance $R_{12,34}$.

Supplementary Note 7: transport and sMIM results of another 6-SL $MnBi_2Te_4$

We show the transport and sMIM results of another 6-SL $MnBi_2Te_4$ device here to complement the 6-SL $MnBi_2Te_4$ experiment reported in the main text. They were all taken at 1.7 K. Supplementary Fig. 7(a) is the optical image of this 6-SL $MnBi_2Te_4$ device. Supplementary Fig. 7(b) shows the magnetic field dependent longitudinal resistance R_{xx} and Hall resistance R_{yx} at +25 V gate voltage, close to its CNP. Both ZHP and the Chern insulator phase are observed similar to the device of the main text. Supplementary Fig. 7(c) shows the field dependent sMIM images at +14 V gate voltage. In addition to the chiral edge state of the Chern insulator phase and the field driven bulk MIT transition, it also unambiguously shows the existence of an edge state at ZHP phase.

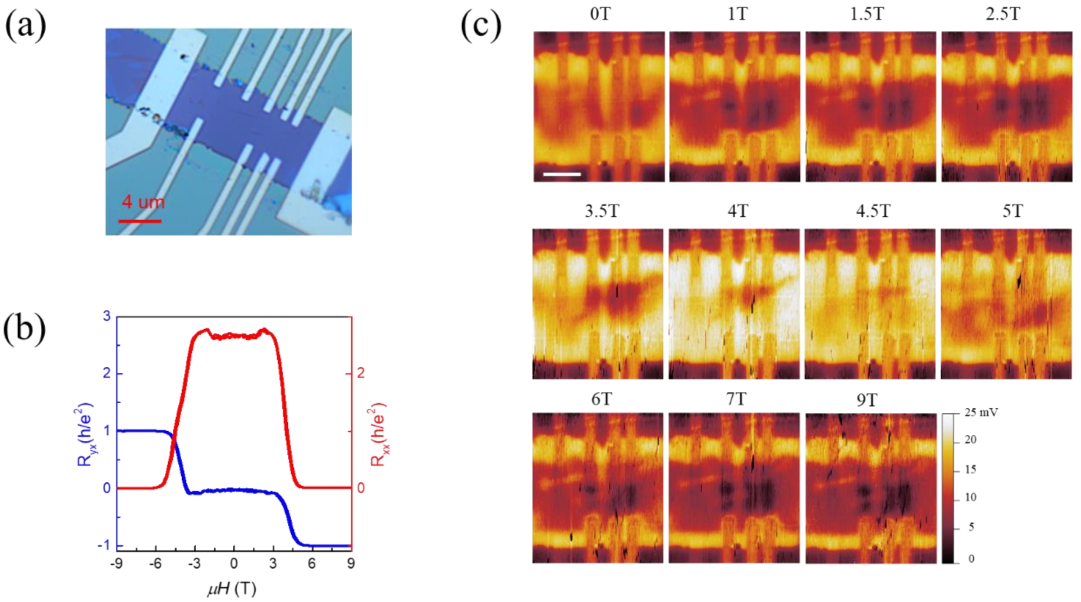

Supplementary Fig. 7 (a) The optical image of another 6-SL $MnBi_2Te_4$ device. (b) The magnetic field dependent R_{xx} and R_{yx}. (c) The field dependent sMIM images. The scale bar is 4 μm.

Supplementary Note 8: model calculations for odd-layer $MnBi_2Te_4$

We adopt the same model as those in Fig. 4 of the main text to calculate the band structure and corresponding wave functions for odd-layer $MnBi_2Te_4$. In Supplementary Fig. 8, one can see ($C=1$) QAH phases with one chiral edge mode for different odd ($N_z = 11, 13, 15$) layer systems. Here the

Chern number *C=1* phase occurs because both the top and bottom surface contribute $e^2/2h$ Hall conductance, when they are in the same magnetization direction.

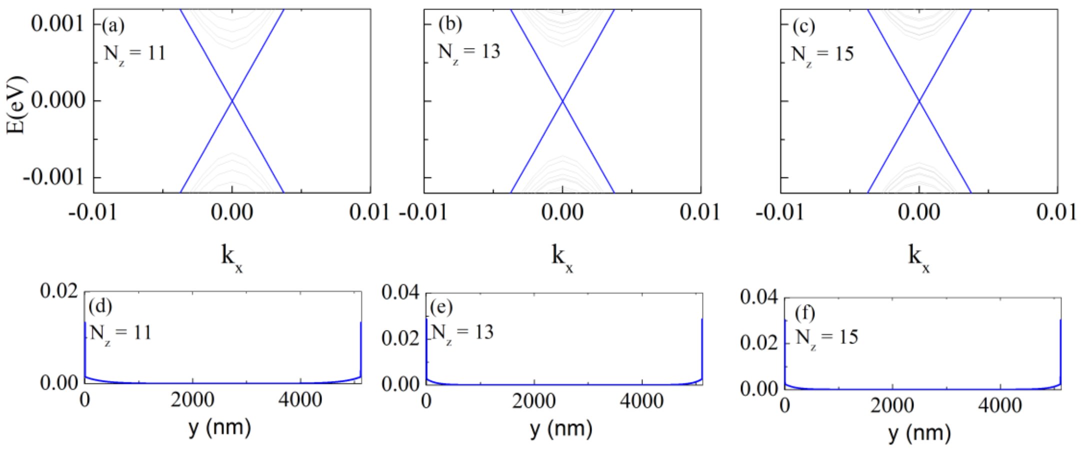

Supplementary Fig. 8 (a-c) Evolution of band structures for different layer number N_z. The blue lines indicate the chiral edge modes. The system employs a periodic boundary condition in the x direction and an open boundary conditions in the y direction. (d-f) spatial distributions of the typical wave functions along y direction at $\mathrm{E} = 0$ for different layer number N_z. The model parameters are the same as those in Fig. 4 of the main text.

Reference:

1 Ovchinnikov, D. *et al.* Intertwined Topological and Magnetic Orders in Atomically Thin Chern Insulator MnBi2Te4. *Nano Lett.* **21**, 2544-2550, (2021).
